# High-Quality Draft Genome Sequence of *Arthrobacter* sp. OY3WO11, a Strain That Inhibits the Growth of *Phytophthora infestans*

**DOI:** 10.1128/genomeA.00585-16

**Published:** 2016-06-23

**Authors:** Jennifer Town, Patrice Audy, Susan M. Boyetchko, Tim J. Dumonceaux

**Affiliations:** a Saskatoon Research and Development Centre, Agriculture and Agri-Food Canada, Saskatoon, Saskatchewan, Canada; b Department of Veterinary Microbiology, University of Saskatchewan, Saskatoon, Saskatchewan, Canada; c Québec Research and Development Centre, Agriculture and Agri-Food Canada, Québec, Québec, Canada

## Abstract

*Arthrobacter* sp. strain OY3WO11 inhibits the growth of the potato pathogen *Phytophthora infestans* in *in vivo* growth challenge assays. We determined the draft genome sequence of this strain, assembling it into 3 scaffolds of 4.2 Mbp total length. OY3WO11 may represent a novel species of *Arthrobacter*.

## GENOME ANNOUNCEMENT

*Arthrobacter* sp. strain OY3WO11 is a Gram-positive bacterium isolated from the rhizosphere of wild oat in Oyen, Alberta, Canada. This organism is a potential biocontrol agent for potato late blight based on its phenotype of disease suppression in *Phytophthora infestans*-challenged plants. To examine the possible mechanisms by which OY3WO11 exhibits this phenotype, we determined its genomic sequence.

*Arthrobacter* sp. OY3WO11 was grown at 22°C in a rotary shaker for 24 to 48 h in yeast extract glucose medium (2.0 g/liter yeast extract, 2.5 g/liter glucose, 0.4 mM MgSO_4_·7H_2_O, 0.09 mM MnSO_4_·H_2_O, 0.85 mM NaCl, 0.017 mM FeSO_4_·7H_2_O, 1.84 mM KH_2_PO_4_, and 1.43 mM K_2_HPO_4_). Genomic DNA was purified from 1 ml of overnight culture using the Wizard genomic DNA (gDNA) extraction kit (Promega, Madison, WI, USA) and sequenced on the GS Junior using Titanium Plus chemistry (Roche Diagnostics, Laval, Quebec, Canada). A total of 142,372 shotgun reads of 644-bp average length was generated. In addition, an 8-kb-insert paired-end sequencing run was performed based on the paired-end rapid library preparation protocol for Titanium chemistry (Roche, March 2012), with modifications as described previously (1). A total of 170,435 paired-end reads was generated, with an estimated pair distance of 6,383 ± 1,595 bp. Assembly of all sequencing runs together produced an improved high-quality draft (2) sequence, with 35× genome coverage. The data were assembled using Newbler version 3.0 (454 Life Sciences), generating 3 scaffolds of 4,253,657 bp (containing 18 contigs), 258,581 bp (1 contig), and 4,841 bp (1 contig). The sequence data were annotated using the Prokaryotic Genome Annotation Pipeline version 3.1 (NCBI).

*Arthrobacter* sp. OY3WO11 had a total genome size of 4,517,079 bp, with a G+C content of 65.29%. Genome annotation revealed 4,225 genes and 4,163 protein-coding genes. The genome featured 1 gene encoding 5S rRNA, 2 genes encoding 16S rRNA, 1 gene encoding 23S rRNA, and 50 tRNA-encoding genes.

The genome of *Arthrobacter* sp. OY3WO11 contained genes that have been associated with biocontrol phenotypes, including phenazine carboxylic acid synthesis (3) and cell wall-degradative enzymes. Two genes encoding putative beta-lactamases were observed. The sequences of taxonomic markers, including the 16S rRNA-encoding gene and *rpoB* (4), shared 97 to 99% identity with the corresponding genes found in *A. phenanthrenivorans* Sphe3. Similarly, two copies of the bacterial barcode marker *cpn60* (5) were identified, each of which clustered with corresponding copies from *A. phenanthrenivorans* Sphe3 by phylogenetic analysis and had sequence identities of 93 to 96%. Comparison of the genome sequence of *Arthrobacter* sp. OY3WO11 to 85 genomic sequences from *Arthrobacter* spp. annotated at the Integrated Microbial Genomes portal (https://img.jgi.doe.gov/cgi-bin/mer/main.cgi) revealed that the average nucleotide identity (ANI) of the genome of OY3WO11 was below the specified cutoff (6) for inclusion in any of the species included in the analysis (the closest ANI was *A. phenanthrenivorans* Sphe3, at 85.25%). In addition, SpecI (7) could not assign OY3WO11 to a species cluster; the closest match was *A. phenanthrenivorans* Sphe3, with an average ANI of 93.9% over 40 Clusters of Orthologous Groups (COGs). Taken together, these observations suggest that OY3WO11 may represent a previously uncharacterized or unsequenced strain of *Arthrobacter*.

### Nucleotide sequence accession numbers.

This whole-genome shotgun project has been deposited at DDBJ/ENA/GenBank under the accession no. LWLP00000000. The version described in this paper is version LWLP01000000.

## References

[1] Hill J, Chaban B, Town J, Links M, Dumonceaux T 2014 Modified paired end rapid library preparation protocol for 454 GS Junior 8-kb library preparation using Covaris g-tubes and BluePippin electrophoresis. Protocol Exch. doi:10.1038/protex.2014.028.

[2] Chain PSG, Grafham DV, Fulton RS, Fitzgerald MG, Hostetler J, Muzny D, Ali J, Birren B, Bruce DC, Buhay C, Cole JR, Ding Y, Dugan S, Field D, Garrity GM, Gibbs R, Graves T, Han CS, Harrison SH, Highlander S, Hugenholtz P, Khouri HM, Kodira CD, Kolker E, Kyrpides NC, Lang D, Lapidus A, Malfatti SA, Markowitz V, Metha T, Nelson KE, Parkhill J, Pitluck S, Qin X, Read TD, Schmutz J, Sozhamannan S, Sterk P, Strausberg RL, Sutton G, Thomson NR, Tiedje JM, Weinstock G, Wollam A, Genomic Standards Consortium Human Microbiome Project Jumpstart Consortium, Detter JC 2009 Genomics. Genome project standards in a new era of sequencing. Science 326:236–237. doi:10.1126/science.1180614. 19815760 PMC3854948

[3] Arseneault T, Goyer C, Filion M 2013 Phenazine production by *Pseudomonas* sp. LBUM223 contributes to the biological control of potato common scab. Phytopathology 103:995–1000. doi:10.1094/PHYTO-01-13-0022-R. 23883153

[4] Adékambi T, Drancourt M, Raoult D 2009 The *rpoB* gene as a tool for clinical microbiologists. Trends Microbiol 17:37–45. doi:10.1016/j.tim.2008.09.008. 19081723

[5] Links MG, Dumonceaux TJ, Hemmingsen SM, Hill JE 2012 The chaperonin-60 universal target is a bar code for bacteria that enables *de novo* assembly of metagenomic sequence data. PLoS One 7:e49755. doi:10.1371/journal.pone.0049755. 23189159 PMC3506640

[6] Richter M, Rosselló-Móra R 2009 Shifting the genomic gold standard for the prokaryotic species definition. Proc Natl Acad Sci USA 106:19126–19131. doi:10.1073/pnas.0906412106. 19855009 PMC2776425

[7] Mende DR, Sunagawa S, Zeller G, Bork P 2013 Accurate and universal delineation of prokaryotic species. Nat Methods 10:881–884. doi:10.1038/nmeth.2575. 23892899

